# A Case Report of Wernicke’s Encephalopathy Disguised As Limbic Encephalitis: A Clinical Puzzle

**DOI:** 10.7759/cureus.28070

**Published:** 2022-08-16

**Authors:** Zaheer A Qureshi, Deny Ponnachan, Haider Ghazanfar, Trishna Acherjee, Faryal Altaf, Manjeet Dhallu

**Affiliations:** 1 Internal Medicine, Icahn School of Medicine at Mount Sinai, New York City, USA; 2 Internal Medicine, BronxCare Health System, Bronx, USA; 3 Neurology, BronxCare Health System, Bronx, USA

**Keywords:** lumbar puncture, alcohol, encephalopathy, altered mental status, neurology, medicine, limbic, encephalitis, korsakoff, wernicke

## Abstract

Wernicke's encephalopathy (WE) is the presence of neurological symptoms in the central nervous system caused by thiamine (Vitamin B1) deficiency. It is an acute clinical condition characterized by confusion, ataxia, and ophthalmoplegia triad. WE is most commonly observed in chronic alcohol users, while it can also present in non-alcoholics. We present a 33-year-old man with alcohol-induced WE who presented with altered mental status and fever. His initial diagnosis was skewed towards bacterial meningitis and limbic encephalitis, but MRI findings were consistent with WE. The patient responded promptly to intravenous (IV) thiamine infusion, and his mental status changed significantly. Repeat EEG in 15 days shows complete recovery with normal brain wave activity. Untreated WE is a significant cause of permanent neurological morbidity and mortality, easily preventable. High suspicion of WE should always be entertained, especially when patients have a known history of alcohol use. Early initiation of IV thiamine could prevent the consequences. Hence, it is essential to raise awareness of WE to take measures without delay and reduce mortality and morbidity with an improved prognosis.

## Introduction

Wernicke's encephalopathy (WE) is the presence of neurological symptoms in the central nervous system caused by thiamine (Vitamin B1) deficiency. It is an acute clinical condition characterized by confusion, ataxia, and ophthalmoplegia triad. Carl Wernicke first reported WE in 1881 [1]. WE is commonly observed in chronic alcohol users. WE may present in the general population with a prevalence of around 2% and is considered underdiagnosed [2]. However, various conditions associated with WE have been described in recent years, such as frequent vomiting, infectious disease, malignancy like Hodgkin lymphoma, bariatric surgery, and celiac disease. WE are a lethal condition; delaying the treatment may result in irreversible dementia and death. Early treatment with intravenous (IV) thiamine can reverse WE, but peripheral nervous system symptoms like neuropathy improve slowly over a period [3,4]. Here, we are discussing a case of a 33-year-old man with alcohol-induced WE, who responded promptly to IV thiamine infusion, and his mentation improved significantly to baseline.

## Case presentation

A 33-year-old man with a fever was admitted with altered mental status (AMS) for two days. The patient had a medical history of hypertension and was compliant with his medications. As per the patient's family, the patient drinks alcohol socially and has not had any such episodes of confusion before. The patient's family denied any history of chills or fever at home. His initial vitals in the emergency department were respiratory rate of 14 breaths per minute, blood pressure of 130/80 mmHg, temperature of 101.5 F, and heart rate of 137 beats per minute. The patient was acutely agitated in the ED. He received IV sedation and was started on prophylactic meningitis treatment given altered mental status and fever. He was later admitted to the intensive care unit (ICU). Physical examination showed the patient was alert but not oriented to person, place, and time. Neurological examination showed no motor or sensory deficit. The patient had gaze palsy with direction-changing nystagmus. Pupil examinations were unremarkable. Examination of other systems revealed no abnormalities.

Initial labs were significant with leukocytosis of 20.8 k/ul, serum sodium of 151 mEq/L, serum blood urea nitrogen (BUN) 16.0 mg/dL, serum creatinine 1.6 mg/dL, serum creatine kinase (CK) 259 unit/L, serum lactic acid of 10.2 mmoles/L, normal serum acetaminophen, and acetylsalicylic acid level. Urine toxicology was positive for benzodiazepines, and serum ethanol level was normal. X-ray chest was unremarkable, and computed tomography (CT) head was unremarkable. HIV antibody test, Rapid Plasma Regain (RPR), West Nile IgM, Vitamin B12 level, thyroid-stimulating hormone (TSH) level, and anti-neutrophil cytoplasmic antibody (ANCA) were negative. Rheumatologic workup includes rheumatoid factor, anti-Jo antibody, anti-ribosomal P antibody, serum anti-topoisomerase antibody, anti-Sjogren's syndrome-related antigen A and B autoantibodies (anti-SSA, anti-SSB), anti-DNA antibody were all negative. All cultures, including blood, urine, and respiratory, are negative with no growth. 

Mental status changes, fever, and leukocytosis were more favorable for an infectious process, and bacterial meningitis was on top of the differential diagnosis. Lumbar puncture (LP) was done given suspected meningitis and was negative for meningoencephalitis. Herpetic encephalitis was part of the differential as well, and IV acyclovir was started. Cerebrospinal fluid analysis showed a white blood cell count of 4, glucose of 42 mg/dL, and protein of 30 mg/dL.

**Figure 1:**
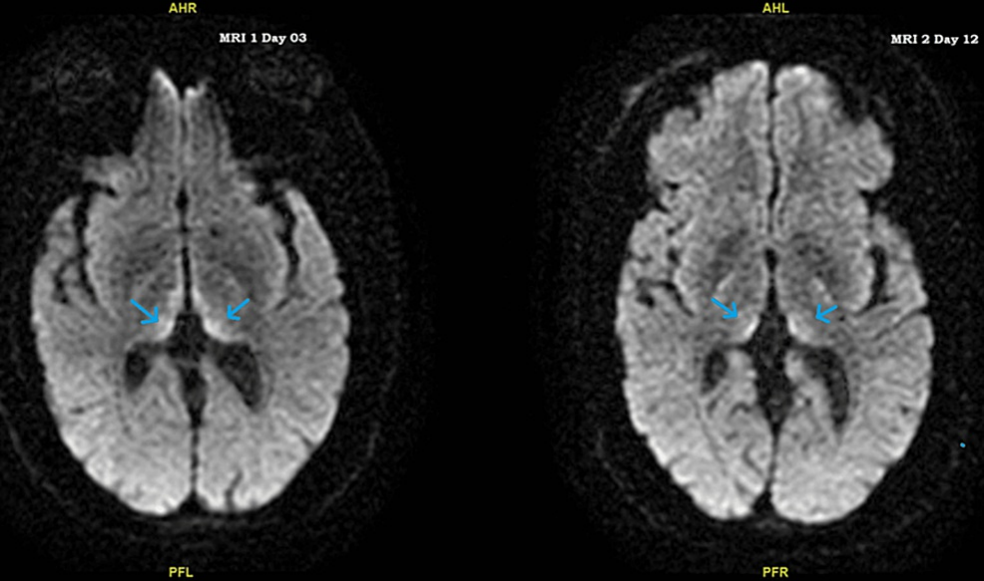
Comparison of MRI brain without contrast on day 03 and day 12. The first MRI shows diffusion-weighted imaging (DWI) showing typical Wernicke's encephalopathy changes in the bilateral medial thalami compared to the second MRI showing improved thalami. Blue arrows show a limbic system

**Figure 2:**
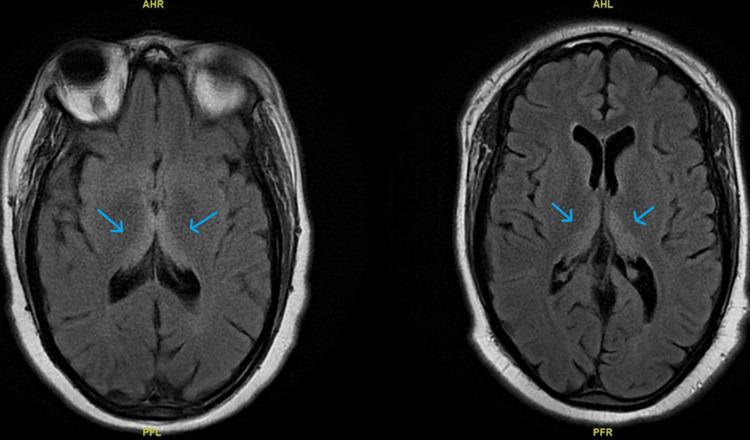
Comparison of MRI brain without contrast on day 03 and day 12. The first MRI shows increased T2-weighted signals in the periaqueductal gray matter and within the medial thalami bilaterally compared to the second MRI showing reduced signals and normal architecture. Blue arrows show a medial thalami

**Figure 3:**
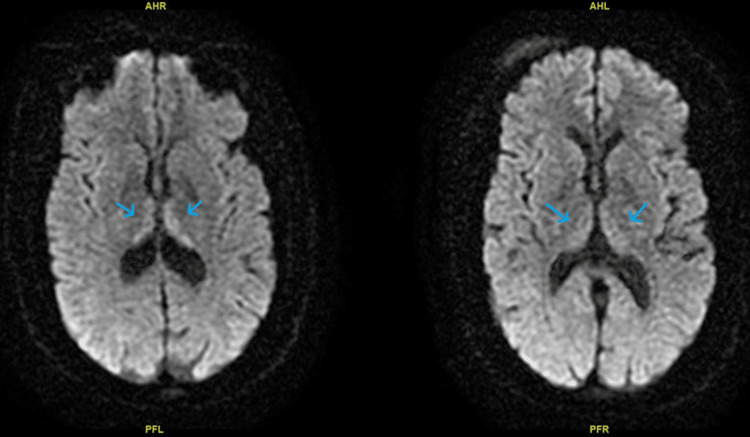
Comparison of MRI brain without contrast on day 03 and day 12. The first MRI shows typical Wernicke's encephalopathy involving medial thalami bilaterally compared to the second MRI showing improved thalami bilaterally. Blue arrows show medial thalami and limbic system

MRI brain without contrast was obtained and was consistent with signal changes in limbic structures and brain stem, raising suspicion for limbic vs. autoimmune encephalitis. The patient was started on a high-dose IV thiamine infusion. The dosing protocol included 500mg thrice daily for five days, then tapered to 250mg once daily for five days. An electroencephalogram (EEG) showed persistent slowing and lack of normal background activity with no triphasic waves, periodic lateralized epileptiform discharges (PLEDs), or other epileptiform activity. The patient continued to be confused, drowsy, and unable to follow commands. The patient also had Gaze palsy with direction-changing nystagmus (horizontal and vertical). Repeat MRI brain after nine days revealed reduced signal changes in bilateral medial thalami and overall normal architecture compared to the first MRI. A comparison between MRI 1 and MRI 2 has been shown in Figures 1-3. EEG was repeated after 15 days, showing marked improvement with faster frequencies and much less slowing than the previous recording. There was no area of prominent dysfunction currently. Over the course, the patient's mentation improved to normal, and he was discharged home.

## Discussion

WE is an acute Neuropsychiatric Emergency caused by the deficiency of thiamine (Vitamin B1), presenting with the classical triad of altered mental status, confusion, ataxia, nystagmus, and ophthalmoplegia [5,6]. In developed nations with dietary supplementing of thiamine, the major cause of deficiency is chronic alcoholism. However, other lesser causes include malnutrition secondary to gastric bypass, hyperemesis gravidarum, anorexia, and other eating disorders, renal replacement therapy, diuretic overuse, hypermetabolic states, thyrotoxicosis, and increased demand in pregnancy and lactation, among a few others [7,8].

Thiamine is an important cofactor for Carbohydrate metabolism and is needed to function enzymes Transketolase, Pyruvate Dehydrogenase, and Alpha-Ketoglutarate dehydrogenase. Although thiamine is utilized by all body cells, the brain and heart are more vulnerable and sensitive to deficiency. Thiamine is also necessary for the maintenance of myelin sheath [8,9] Alcohol results in thiamine deficiency mainly by decreased dietary intake in alcoholics, alcohol interfering with the uptake of thiamine from the gastrointestinal tract by inhibiting transport carrier molecules, and concomitant deficiency of magnesium in chronic alcoholics resulting in impaired function of thiamine utilizing enzymes [9,10]. Low thiamine levels have been reported in 30%-80% of patients with alcohol use disorder. The extent of deficiency varies based on malnutrition, extent, duration of alcohol intake, and liver damage [11].

Studies in rats by Xe et al. showed significant brain abnormalities in the alcohol/pyrithiamine (thiamine antagonist) group compared to the alcohol exposed-Thiamine replete group showing that Thiamine deficiency was the causal agent for WE [12]. The mechanism of neuronal damage by inhibiting the enzymes mentioned above is via focal lactic acidosis, cerebral energy impairment, excess glutamate causing depolarization, and NMDA-mediated excitotoxicity. Other mechanisms may include free radical-mediated damage and interference with the blood-brain barrier [13]. The primary sites of the brain involved are periaqueductal gray matter, mamillary bodies, medial thalamus, and superior cerebellar vermis. This results in the typical symptoms of WE [14].

WE is primarily a clinical diagnosis. A high degree of clinical suspicion is necessary for prompt treatment. Detailed patient history, including alcohol use, lab studies, and neuroimaging, can provide additional help. Our patient presented with altered mental status and the MRI showed hyperintensity of the temporal lobes bilaterally on T2.

A post-mortem brain tissue analysis study by Harper et al. showed that 80% of patients were not correctly diagnosed [15]. Numerous studies have shown that the classic triad is present in only one-third of the patient population, and in most cases, only one or two of the triad were present. The most common presenting symptom was Altered Mental Status (82%) and less commonly ataxia (23%), ocular symptoms (29%), and polyneuropathy (11%) [15,16]. WE is diagnosed with patients having two or more of Caine's Criteria [13]. Dietary deficiency, oculomotor abnormality, cerebellar dysfunction/ataxia, altered mental status, and mild memory impairment Caine's Criteria showed a higher antemortem diagnosis than the classical triad [17].

Whole blood thiamine level by high-performance liquid chromatography - commonly used, easy availability, and tells us about decreased blood levels. Erythrocyte thiamine transketolase levels before and after administering thiamine - 25% increase in confirmatory [18]. Both CT and MRI imaging can be used. However, MRI has a much higher sensitivity in helping confirm the diagnosis and can be considered the imaging of choice. CT is not a reliable source for aiding in diagnosis. MRI shows a high-intensity signal on T2-weighted images in medial thalamic and periaqueductal regions in the acute stage of WE. In late stages, atrophy of mammillary bodies and cerebellum with third ventricular dilation is more prominent [19-21].

A high degree of clinical suspicion is required when a patient presents atypical symptoms or incomplete triad of confusion, ataxia, and ophthalmoplegia, especially with a background of alcohol use, to promptly diagnose and treat WE [15]. There are no universally accepted thiamine dosage guidelines for treating this disorder. The only consensus is early treatment with high doses of thiamine [22]. EFNS Guidelines say thiamine should be administered 200mg three times a day for two to three days, preferably as an IV infusion for non-alcoholics [6]. It should also be given prior to any carbohydrate administration. Additionally, 250-1,000 mg/day should be administered orally. Treatment should be continued with oral therapy until no further clinical improvement or complete resolution of symptoms occur [23]. In patients with uncomplicated alcohol dependence without symptoms, oral thiamine 250-500 mg/day can be given for three to five days, followed by 100-250 mg/day. A high dose of thiamine (>500 mg) is safe and efficacious in alcohol users with suspected WE. In a case series by Nishimoto et al., patients with suspected WE were treated with ≥500 mg of thiamine for an average of three days; 73% showed resolution or improvement of symptoms after treatment [24]. The half-life of intravenously administered thiamine is 96 minutes. Hence, it is only imperative to administer it in divided doses daily [25].

Undiagnosed/untreated WE is a major cause of easily preventable permanent neurological morbidity and mortality [26]. Even if there is a small probability of a patient having WE, treatment should not be delayed until confirmation of diagnosis. Clinicians should have a low threshold for treatment with thiamine due to its easy availability, good safety profile, and inexpensive nature. Urgent treatment is necessary to avoid neuronal cell death or disease progression into irreversible Korsakoff syndrome. This rate can be as high as 85% in untreated patients, and mortality as high as 20% [27]. Iatrogenic precipitation of WE can be avoided by always administering thiamine prior to dextrose, especially with patients presenting with at least one of the clinical criteria in a background of alcohol use [6]. Preventive strategies include thiamine fortification of daily food items. A study from Australia showed a significant reduction in WE after the fortification of bread flour. The study compared the prevalence of the disease via brain autopsy after fortification was introduced with that of prevalence from a similar study [28]. Other preventive measures include treating patients with alcohol withdrawal with oral thiamine during hospital admission and oral prophylactic therapy for alcohol dependence [29].

## Conclusions

Undiagnosed and untreated WE is a major cause of easily preventable permanent neurological morbidity and mortality. Even if there is a small probability of having WE, treatment should not be delayed until confirmation of diagnosis. This case report illustrates the diverse complexity of patients with altered mental status. Even though it is low in differentials, high suspicion of WE should be considered, especially when alcoholics are considered. Although prevalent in alcoholics, WE can occur in anyone with nutritional thiamine deficiency, including non-alcoholics. In these cases, early initiation of IV thiamine should be started. As physicians, we should probably keep a low threshold for the diagnosis, which may lead to devastating consequences without early treatment, leading to devastating consequences. Hence, it is essential to raise awareness of WE to take measures without delay and reduce mortality with an improved prognosis. Early brain MRI should be part of any altered mental status patient workup.
